# Joint Associations of Race, Ethnicity, and Socioeconomic Status With Mortality in the Multiethnic Cohort Study

**DOI:** 10.1001/jamanetworkopen.2022.6370

**Published:** 2022-04-11

**Authors:** Meera Sangaramoorthy, Salma Shariff-Marco, Shannon M. Conroy, Juan Yang, Pushkar P. Inamdar, Anna H. Wu, Christopher A. Haiman, Lynne R. Wilkens, Scarlett L. Gomez, Loïc Le Marchand, Iona Cheng

**Affiliations:** 1 Department of Epidemiology and Biostatistics, School of Medicine, University of California, San Francisco; 2 Helen Diller Family Comprehensive Cancer Center, University of California, San Francisco; 3 Department of Public Health Sciences, University of California, Davis; 4 Department of Preventive Medicine, Keck School of Medicine, University of Southern California, Los Angeles; 5 Epidemiology Program, University of Hawaii Cancer Center, Honolulu

## Abstract

**Importance:**

Socioeconomic status may help delineate racial and ethnic inequities in mortality.

**Objective:**

To investigate the joint associations of race, ethnicity, and neighborhood and individual socioeconomic status with mortality.

**Design, Setting, and Participants:**

This prospective analysis used data from the Multiethnic Cohort Study. A population-based sample of participants recruited from California (mainly Los Angeles County) and Hawaii from 1993 to 1996 was followed up until 2013. African American, European American, Japanese American, Latino American, and Native Hawaiian men and women were included. Participants with baseline residential addresses that could not be geocoded or who were missing information on education or adjustment variables were excluded. Data analyses were conducted from January 2018 to December 2020.

**Exposures:**

Neighborhood socioeconomic status (nSES) was derived using US Census block group data on education, occupation, unemployment, household income, poverty, rent, and house values. Participants self-reported their highest education attainment. Five racial and ethnic groups, 2 states of residence, 2 nSES, and 2 education categories were combined to create a joint exposure variable. Low and high nSES were defined as quintiles 1 to 3 and 4 to 5, respectively. Low and high education levels were defined as high school or less and greater than high school graduate, respectively.

**Main Outcomes and Measures:**

All-cause, cardiovascular disease (CVD), cancer, and non-CVD and noncancer deaths were ascertained through 2013 via linkage to death certificates and the US National Death Index. Multivariable Cox proportional hazards regression analyses were conducted.

**Results:**

Among 182 912 participants (100 785 [55.1%] women and 82 127 [44.9%] men; mean [SD] age, 60.0 [8.9] years; 31 138 African American, 45 796 European American, 52 993 Japanese American, 39 844 Latino American, and 13 141 Native Hawaiian participants) with a mean (SD) follow-up of 17 (5) years, there were 63 799 total deaths, including 23 191 CVD deaths, 19 008 cancer deaths, and 21 235 non-CVD and noncancer deaths. The lowest all-cause mortality was found among 15 104 Japanese American participants in Hawaii with high nSES and high education (eg, 2870 all-cause deaths [19.0%]), and this population served as the reference group for all regression analyses. Native Hawaiian participants in Hawaii with low nSES and low education had the highest all-cause mortality HR (2.38; 95% CI, 2.21-2.57). African American and European American participants in California with low nSES and low education had the next highest all-cause mortality HRs (2.01; 95% CI, 1.91-2.11 and 1.98; 95% CI, 1.85-2.12, respectively). Latino American participants in California with low nSES had equivalent all-cause mortality HRs regardless of education level (high education: 1.57; 95% CI, 1.48-1.66; low education: 1.57; 95% CI, 1.50-1.65). Patterns for cause-specific mortality were similar to those for all-cause mortality. For example, Native Hawaiian participants in Hawaii with low nSES and low education had highest CVD mortality HR (2.92; 95% CI, 2.60-3.27) and cancer mortality HR (2.01; 95% CI, 1.77-2.29).

**Conclusions and Relevance:**

These results suggest that joint associations of nSES and education may further delineate racial and ethnic inequities in mortality and that future investigations of racial and ethnic inequities in mortality should consider differences by measures of socioeconomic status, especially for underserved populations.

## Key Points

**Question:**

Do joint associations of race, ethnicity, and socioeconomic status further delineate racial and ethnic inequities in mortality?

**Findings:**

In this cohort study of 182 912 African American, European American, Japanese American, Latino American, and Native Hawaiian adults in California and Hawaii, Native Hawaiian participants in Hawaii with low neighborhood socioeconomic status and low education levels experienced the highest all-cause and cause-specific mortality hazard ratios, with significant mortality increases compared with the reference group of Japanese American participants in Hawaii with high neighborhood socioeconomic status and high education levels.

**Meaning:**

These results suggest that joint examination of race, ethnicity, and socioeconomic status may refine investigations of mortality inequities.

## Teaser

This cohort study examines the joint associations of race, ethnicity, and socioeconomic status with all-cause, cardiovascular disease, cancer, and other mortality among a racially and ethnically diverse group of adults in 2 US states.

## Introduction

Although mortality rates in the US have steadily declined since 1970,^1^ significant inequities by race and ethnicity persist, with African American populations experiencing the highest mortality, followed by European American, Latino American, and Asian American and Pacific Islander populations.^2^ Disaggregation of the Asian American and Pacific Islander population reveals a higher mortality rate among Native Hawaiian populations compared with European American populations.^3,4^ Similar patterns of racial and ethnic inequities exist for heart disease and cancer mortality, the 2 leading causes of death in the US.^5^

Social determinants of health are associated with racial and ethnic inequities in morbidity and mortality.^6,7^ Structural racism, acting through policies and practices of multiple societal institutions and systems, including housing, education, employment, health care, and criminal justice,^8,9^ is associated with the unequal distribution of resources and socioeconomic status (SES) across racial and ethnic groups. Understanding the interplay of race, ethnicity, and SES provides a critical approach to investigate the unequal burden of mortality across populations.^6,7,10^

Neighborhood-level SES (nSES; based on education, occupation, employment, income, poverty, and home values in a geographical area) and individual-level SES (often measured as personal or household income or educational attainment) have been inversely associated with all-cause and cause-specific mortality.^11,12,13,14,15,16,17,18^ Examination of the joint associations of nSES and individual SES with mortality has elucidated patterns not otherwise seen when these factors are considered separately.^11,12,13,14,15,16^ However, associations have not been consistent. In some studies,^11,12,13,16^ mortality was highest among participants with low nSES and low individual SES, whereas in other studies,^14,15^ mortality was highest for participants with high nSES and low individual SES. Moreover, these previous analyses included relatively small study populations with limited racial and ethnic diversity.

Considering the limitations of previous studies, we sought to investigate the joint associations of race, ethnicity, state of residence, nSES, and education with all-cause and cause-specific mortality for 182 912 participants in the Multiethnic Cohort (MEC) Study. Moving beyond traditional research methods that focus solely on racial and ethnic differences in mortality,^6,7,19^ we hypothesized that our approach of jointly considering socioeconomic status with race and ethnicity as informed by an intersectional perspective^20,21^ would further delineate racial and ethnic inequities in mortality. Furthermore, the MEC’s inclusion of racial and ethnic groups often underrepresented in epidemiologic investigations, such as African American, Japanese American, Latino American, and Native Hawaiian populations,^22^ may make an important contribution to identifying and quantifying racial and ethnic inequities in mortality.

## Methods

Receipt of a completed, mailed baseline questionnaire was considered implicit consent to participate in the MEC by the institutional review boards (IRBs) of the University of Hawaii and the University of Southern California. This cohort study was approved by these IRBs, in addition to that of the University of California, San Francisco, and all participating sites received a waiver of consent per IRB policy. This study followed the Strengthening the Reporting of Observational Studies in Epidemiology (STROBE) reporting guideline for cohort studies.

### Study Population

The MEC is a population-based cohort of African American, European American, Japanese American, Latino American, and Native Hawaiian men and women aged 45 to 75 years at recruitment in 1993 to 1996. The study population was described in detail previously.^23^ Potential participants from Hawaii and mainly Los Angeles County in California were identified primarily through driver license files, as well as Health Care Financing Administration files in California and voter registration files in Hawaii. Eligible participants were mailed a 26-page baseline questionnaire assessing demographics, anthropometrics, medical history, family history, reproductive history, physical activity, and diet. Study eligibility, enrollment, and exclusions are illustrated in eFigure 1 in the Supplement. Of more than 850 000 eligible participants,^24^ 215 251 individuals completed the baseline questionnaire and enrolled in the study. Most participants (194 835 individuals [90.5%]) self-reported 1 race or ethnicity in the baseline questionnaire (eFigure 2 in the Supplement). Participants who self-reported more than 1 race or ethnicity were assigned to 1 of 5 racial and ethnic categories eligible for inclusion in the MEC (which was funded to recruit these specific groups) in the following order: African American, Native Hawaiian, Latino American, Japanese American, and European American.

For this study, we excluded 15 406 participants with baseline residential addresses that could not be geocoded, 8160 participants with missing adjustment variables, 3135 participants with missing baseline nSES and education data, 4049 participants who did not self-identify as 1 of the 5 eligible racial and ethnic groups, 1580 Cuban or Caribbean Latino American participants for whom diet could not be adequately assessed, and 9 participants with no follow-up. The final analysis included 182 912 participants.

### nSES and Education

Geocoding of participant baseline residential addresses (1993-1996) and construction of an nSES index have been previously described.^25,26,27^ The nSES index is an established composite measure created by principal component analysis of US Census data that incorporates census block group data on education, occupation, unemployment, household income, poverty, rent, and house values.^28^ Geocoded addresses were linked to 1990 US Census block groups for each state and categorized into quintiles based on the nSES distribution of Los Angeles County and Hawaii block groups for participants in California and Hawaii, respectively. Although parameters of principal component analysis (eg, eigenvalues, eigenvector loadings, and proportion variance) for the nSES index in California and Hawaii were comparable, we detected variation in the distribution of education, poverty, blue collar occupation (as defined in the 1990 census^29^), unemployment, and rent between states, especially for areas of low nSES (eTable 1 in the Supplement). Low and high nSES were defined as quintiles 1 to 3 and 4 to 5, respectively. Most MEC participants in our analysis (143 962 individuals [78.7%]) remained at the same level of nSES between baseline and death or censor. Educational attainment was self-reported as high school graduate or less, vocational school or some college, college graduate, or graduate and professional school. Low and high education were defined as high school or less and greater than high school graduate, respectively.

### Outcome Assessment

Deaths were ascertained through 2013 via linkage to state death certificates and the US National Death Index. Cause-specific deaths were based on the *International Classification of Diseases, Ninth Revision *(*ICD-9*) and *International Statistical Classification of Diseases and Related Health Problems, Tenth Revision *(*ICD-10*). CVD-specific deaths were defined as *ICD-9* codes 390 to 398, 402, 404, 410 to 434, and 436 to 438 and *ICD-10* codes I00 to I09, I11, I13, I20 to I51, and I60 to I69. Cancer-specific deaths were defined as *ICD-9* codes 140 to 239 and *ICD-10* codes C00 to C97 and D00 to D48. We grouped all non-CVD and noncancer deaths into 1 outcome category.

### Statistical Analysis

Follow-up time was calculated from age at cohort entry to age at death or end of follow-up (December 31, 2013). We combined race, ethnicity, state of residence, and high or low nSES and education categories to create 1 joint exposure variable with 28 levels (eg, Japanese American participants in Hawaii with high nSES and high education and Japanese American participants in Hawaii with low nSES and high education). Hazard ratios (HRs) and 95% CIs were calculated for all-cause, CVD-specific, cancer-specific, and non-CVD and noncancer–specific mortality using Cox proportional hazards models with age as the time scale. Deaths from other causes were censored in cause-specific analyses. Models included strata variables of age at cohort entry (<50, 50-54, 55-59, 60-64, 65-69, 70-74, or ≥75 years) and sex (men or women) and were additionally adjusted for the following confounders associated with the joint exposure variable and mortality in the study: marital status (married; single; separated, divorced, or widowed; or unknown), smoking status (accounting for smoking cessation over time^30,31,32^), body mass index (BMI; calculated as weight in kilograms divided by height in meters squared; <18.5, 18.5-22.9, 23.0-24.9, 25.0-29.9, 30.0-34.9, ≥35.0, or unknown), level of vigorous physical activity (men: <0.10, 0.10-0.24, 0.25-0.79, ≥0.80, or unknown h/d; women: <0.10, 0.10-0.24, 0.25-0.49, ≥0.50, or unknown h/d), coffee consumption^32^ (none, 1-3 cups/mo, 1-6 cups/wk, 1 cup/d, >1 cup/d, or unknown), alcohol consumption (men: 0, 1.0-5.1, 5.2-22.9, or ≥23.0 g/d; women: 0, 1.0-2.4, 2.5-9.9, or ≥10 g/d), total energy intake (<1317, 1317-1717, 1718-2159, 2160-2835, or ≥2836 kcal/d), percent energy from fat (<23.7%, 23.7%-28.0%, 28.1%-31.8%, 31.9%-35.9%, or ≥36.0%), and preexisting chronic disease at baseline (self-reported heart attack or angina, stroke, diabetes, or high blood pressure, and cancer that was self-reported or ascertained from tumor registries). Formal mediation analysis was beyond the scope of this study. HR estimates from models with and without potential mediators (such as smoking status, BMI, alcohol consumption, diet, physical activity level, and preexisting chronic conditions) were not appreciatively different, suggesting that mediation was not likely. Sensitivity analyses excluding participants with preexisting chronic conditions were conducted. We conducted subgroup analysis by sex. Heterogeneity across sex was tested using Wald statistics for cross-product terms of the joint exposure variable and sex. Models accounted for clustering by block group using a sandwich estimator of the covariance structure that accounted for intracluster dependence.^33^ All analyses were performed using SAS statistical software version 9.4 (SAS Institute), and *P* values were 2-sided with statistical significance defined as *P* < .05. Data were analyzed from January 2018 to December 2020.

## Results

Among 182 912 participants (100 785 [55.1%] women and 82 127 [44.9%] men; mean [SD] age, 60.0 [8.9] years; 31 138 African American, 45 796 European American, 52 993 Japanese American, 39 844 Latino American, and 13 141 Native Hawaiian participants) with a mean (SD) follow-up of 17 (5) years, there were 63 799 total deaths, including 23 191 CVD deaths, 19 008 cancer deaths, and 21 235 non-CVD and noncancer deaths. Participant characteristics are presented in the Table and eFigures 3 and 4 in the Supplement.

**Table.** Characteristics of Participants by Race and Ethnicity

Characteristic	Race and ethnicity, No. (%) (N = 182 912)				
	African American (n = 31 138)	European American (n = 45 796)	Japanese American (n = 52 993)	Latino American (n = 39 844)	Native Hawaiian (n = 13 141)
Deaths					
All cause	15 081 (48.2)	15 564 (33.9)	16 002 (30.1)	12 602 (31.3)	4550 (34.5)
CVD	6133 (19.7)	5175 (11.3)	5568 (10.5)	4625 (11.6)	1690 (12.9)
Cancer	4445 (14.3)	4847 (10.6)	4703 (8.9)	3652 (9.2)	1361 (10.4)
Non-CVD and noncancer	4377 (14.1)	5500 (12.0)	5669 (10.7)	4203 (10.6)	1486 (11.3)
Age, mean (SD), y^a^	61.3 (9.0)	59.1 (9.0)	61.1 (9.1)	59.8 (7.7)	56.5 (8.6)
State of residence^a^					
Hawaii	0	32 025 (69.9)	40 975 (77.3)	0	12 981 (98.8)
California	31 138 (100)	13 772 (30.1)	12 018 (22.7)	39 844 (100)	160 (1.2)
Sex^a^					
Women	20 082 (64.5)	24 761 (54.1)	27 964 (52.8)	20 572 (51.6)	7406 (56.4)
Men	11 056 (35.5)	21 035 (45.9)	25 029 (47.2)	19 272 (48.4)	5735 (43.6)
Marital status^a^					
Married	14 068 (45.2)	30 706 (67.1)	40 575 (76.6)	26 785 (67.2)	9080 (69.1)
Single	2096 (6.7)	3112 (6.8)	3771 (7.1)	2434 (6.1)	771 (5.9)
Separated, divorced, or widowed	14 515 (46.6)	11 700 (25.6)	8443 (15.9)	10 237 (25.7)	3224 (24.5)
Unknown	459 (1.5)	278 (0.6)	204 (0.4)	388 (1.0)	66 (0.5)
Smoking status^a^					
Never	12 190 (39.2)	18 089 (39.5)	26 844 (50.7)	20 271 (50.9)	5217 (39.7)
Former	7167 (23)	7555 (16.5)	6224 (11.7)	5647 (14.2)	2964 (22.6)
Current	11 781 (37.8)	20 152 (44.0)	19 925 (37.6)	13 926 (35.0)	4960 (37.7)
Preexisting illness^a^^,^^b^	20 845 (67.0)	19 526 (42.6)	26 930 (50.8)	19 629 (49.3)	7317 (55.7)
BMI, mean (SD)^a^	28.5 (5.8)	26.2 (5.0)	24.4 (3.7)	27.7 (4.8)	29.0 (6.2)
Unknown	1085 (3.5)	245 (0.5)	301 (0.6)	398 (1.0)	206 (1.6)
Vigorous physical activity, mean (SD), h/d^a^	0.3 (0.7)	0.4 (0.8)	0.3 (0.7)	0.4 (0.9)	0.6 (1.1)
Unknown	2263 (7.3)	1527 (3.3)	1654 (3.1)	2632 (6.6)	414 (3.2)
Coffee intake, mean (SD), g/d^a^	225.9 (258.8)	373.4 (328.9)	322.5 (290.5)	379.0 (345.8)	277.8 (301.8)
Unknown	1359 (4.4)	759 (1.7)	534 (1.0)	1515 (3.8)	25 (0.2)
Alcohol intake, mean (SD), g/d^a^	7.8 (26.1)	13.8 (27.9)	6.1 (18.5)	8.1 (26.1)	9.1 (27.5)
Energy intake, mean (SD), kcal/d^a^	2019.3 (1084.9)	2064.3 (848.0)	2045.5 (794.5)	2447.2 (1325.0)	2595.9 (1329.7)
Energy from fat, mean (SD), %^a^	32.5 (7.3)	30.5 (7.0)	27.8 (6.5)	31.5 (6.7)	29.7 (6.8)
nSES, quintile^a^					
1 (lowest)	12 967 (41.6)	4625 (10.1)	4597 (8.7)	13 633 (34.2)	2087 (15.9)
2	7343 (23.6)	5679 (12.4)	6860 (13.0)	10 057 (25.2)	2583 (19.7)
3	4896 (15.7)	8786 (19.2)	10 307 (19.5)	7581 (19.0)	2416 (18.4)
4	3998 (12.8)	10 586 (23.1)	11 373 (21.5)	5134 (12.9)	2582 (19.7)
5 (highest)	1934 (6.2)	16 120 (35.2)	19 856 (37.5)	3439 (8.6)	3473 (26.4)
Education^a^					
High school graduate or less	13 080 (42.0)	12 285 (26.8)	21 126 (39.9)	27 577 (69.2)	6939 (52.8)
Vocational school or some college	11 309 (36.3)	14 350 (31.3)	15 692 (29.6)	8342 (20.9)	3912 (29.8)
College graduate	3546 (11.4)	8760 (19.1)	9477 (17.9)	1941 (4.9)	1311 (10.0)
Graduate or professional school	3203 (10.3)	10 401 (22.7)	6698 (12.6)	1984 (5.0)	979 (7.4)
Joint nSES and education^a^^,^^c^					
Low nSES and low education	11 827 (38.0)	6540 (14.3)	10 300 (19.4)	23 313 (58.5)	4160 (31.7)
Low nSES and high education	13 379 (43.0)	12 550 (27.4)	11 464 (21.6)	7958 (20.0)	2926 (22.3)
High nSES and low education	1253 (4.0)	5745 (12.5)	10 826 (20.4)	4264 (10.7)	2779 (21.1)
High nSES and high education	4679 (15.0)	20 961 (45.8)	20 403 (38.5)	4309 (10.8)	3276 (24.9)

Abbreviations: BMI (calculated as weight in kilograms divided by height in meters squared), body mass index; CVD, cardiovascular disease; nSES, neighborhood socioeconomic status.

^a^ Baseline.

^b^ Preexisting illness included heart attack or angina, stroke, diabetes, or high blood pressure that was self-reported and cancer that was self-reported or ascertained from tumor registries before baseline.

^c^ Low and high nSES were defined as quintiles 1 to 3 and 4 to 5, respectively. Low and high education were defined as high school or less and greater than high school graduate, respectively.

African American participants had the highest proportion of all-cause deaths (15 081 individuals [48.2%]), were most likely to be female (20 082 [64.5%] women) and have preexisting conditions (20 845 individuals [67.0%]), and had the lowest mean (SD) coffee intake (225.9 [258.8] g/d). Additionally, they had the highest proportion of individuals in the lowest quintile of nSES (12 967 individuals [41.6%]). European American participants were more likely to have graduate or professional degrees (10 401 individuals [22.7%]) and be current smokers (20 152 individuals [44.0%]) and had the highest mean (SD) alcohol intake (13.8 [27.9] g/d). Japanese American participants had the lowest proportion of deaths from any cause (16 002 individuals [30.1%]), were most likely to be married (40 575 individuals [76.6%]), and had the lowest mean (SD) BMI (24.4 [3.7]) and alcohol intake (6.1 [18.5] g/d). They also had the highest proportion of individuals in the highest quintile of nSES (19 856 individuals [37.5%]). Latino American participants had the highest proportion of individuals with high school degrees or less (27 577 individuals [69.2%]). Native Hawaiian participants were the youngest (mean [SD] age, 56.5 [8.6] years) and had the highest mean (SD) level of vigorous physical activity (0.6 [1.1] h/d).

**Figure 1.**
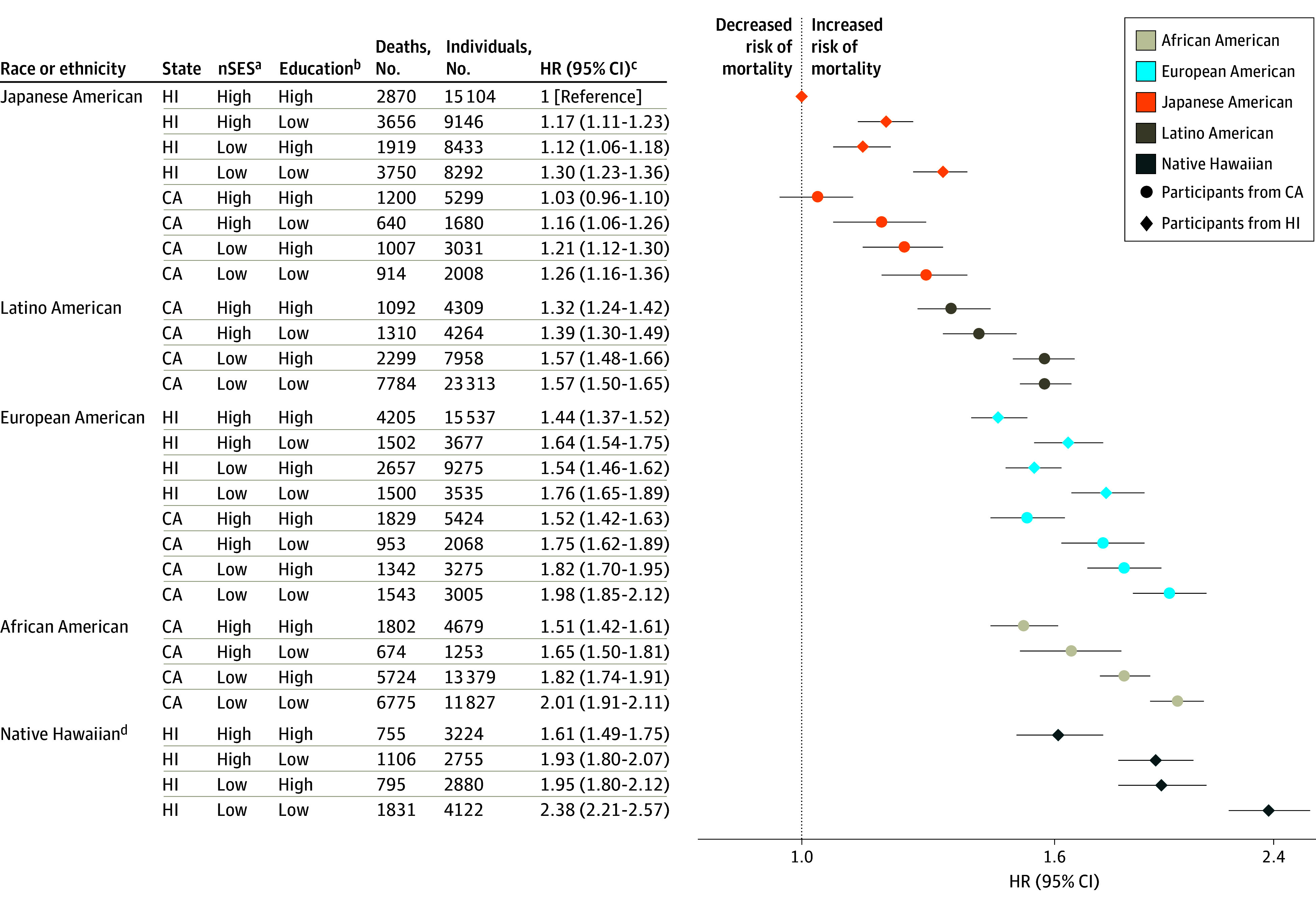
Joint Associations of Factors With All-Cause Mortality Joint associations of race, ethnicity, state of residence, neighborhood socioeconomic status (nSES), and education with mortality are shown. ^a^Low nSES was defined as quintiles 1 to 3 and high nSES as quintiles 4 to 5. ^b^Low education was defined as high school graduate or less and high education as greater than high school graduate. ^c^Model was adjusted for age at cohort entry and sex (as strata variables), marital status, smoking, body mass index (calculated as weight in kilograms divided by height in meters squared), vigorous physical activity level, coffee intake, alcohol intake, total energy intake, percent energy from fat, and preexisting chronic disease. ^d^Native Hawaiian participants living in California are not shown owing to small numbers (160 individuals with 55 deaths).

Figure 1 presents joint associations of race, ethnicity, state of residence, nSES, and education with all-cause mortality. Japanese American participants in Hawaii with high nSES and high education had the lowest mortality (eg, 2870 all-cause deaths among 15 104 individuals [19.0%]) and served as the reference group for all subsequent regression models. In comparison with this reference group, all racial and ethnic groups had statistically significantly increased all-cause mortality (eg, HR for Native Hawaiian participants in Hawaii with high nSES and high education, 1.61; 95% CI, 1.49-1.75); the highest all-cause mortality HR was among Native Hawaiian participants in Hawaii with low nSES and low education (HR, 2.38; 95% CI, 2.21-2.57). African American participants in California with low nSES and low education (HR, 2.01; 95% CI, 1.91-2.11) and European American participants in California with low nSES and low education (HR, 1.98; 95% CI, 1.85-2.12) had the next highest all-cause mortality HRs. Among European American participants in Hawaii, those with low nSES and low education had the highest HR (1.76; 95% CI, 1.65-1.89), followed by those with high nSES and low education (HR, 1.64; 95% CI, 1.54-1.75). Mortality was increased 1.57-fold vs the reference group for Latino American participants in California with low nSES, regardless of education (high education: HR, 1.57; 95% CI, 1.48-1.66; low education: HR, 1.57; 95% CI, 1.50-1.65). Additionally, Japanese American participants with low nSES and low education had similar HRs by state (Hawaii: HR, 1.30; 95% CI, 1.23-1.36; California: HR, 1.26; 95% CI, 1.16-1.36). Among Japanese Americans in Hawaii and European Americans in Hawaii, mortality HRs were higher for those with high nSES and low education than those with low nSES and high education. Considering race and ethnicity alone, compared with Japanese American participants, African American and Native Hawaiian participants had similar all-cause mortality HRs (1.93; 95% CI, 1.89-1.98 and 1.96; 95% CI, 1.90-2.03, respectively) (eTable 2 in the Supplement).

**Figure 2.**
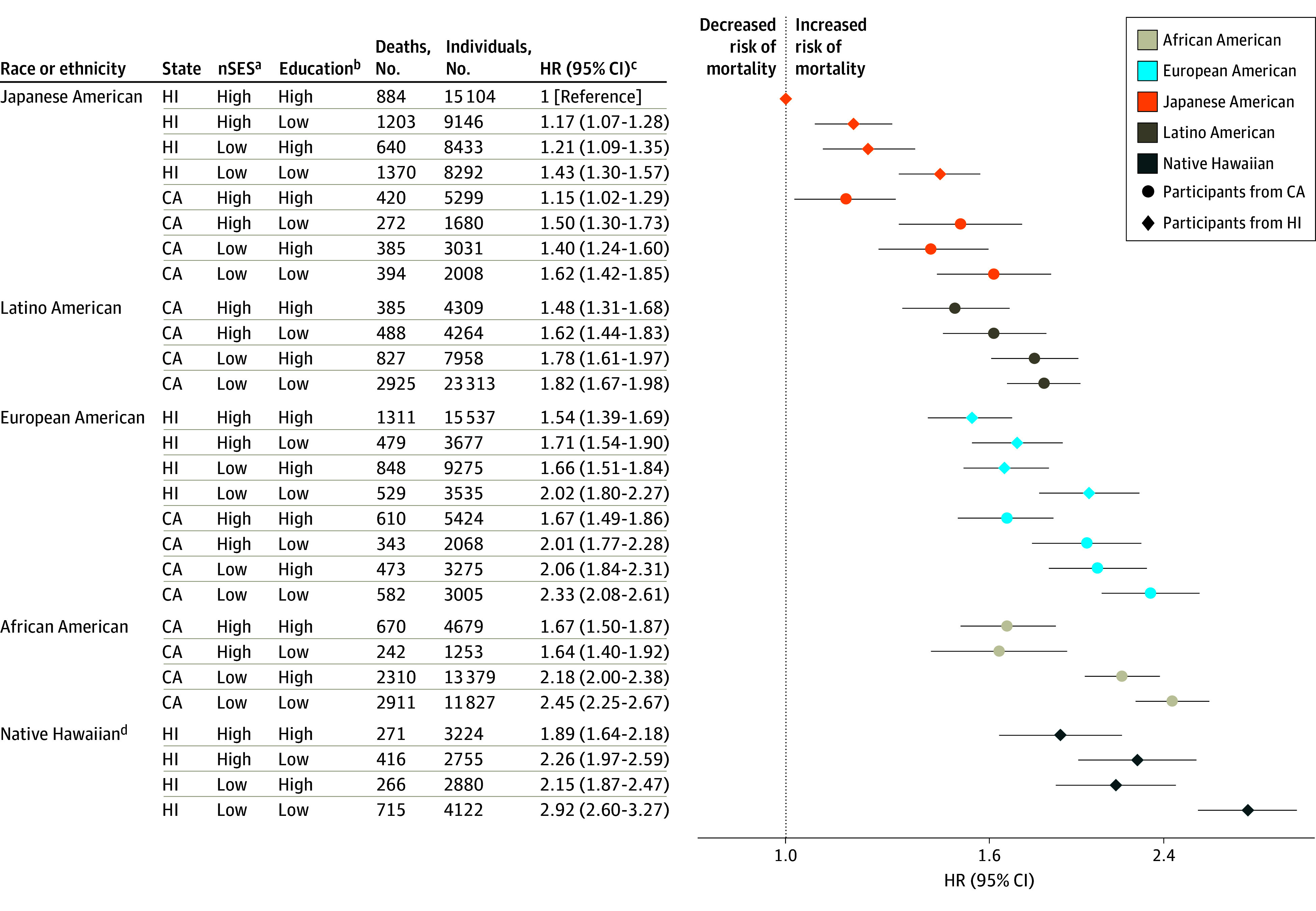
Joint Associations of Factors With Cardiovascular Disease Mortality Joint associations of race, ethnicity, state of residence, neighborhood socioeconomic status (nSES), and education with mortality are shown. ^a^Low nSES was defined as quintiles 1 to 3 and high nSES as quintiles 4 to 5. ^b^Low education was defined as high school graduate or less and high education as greater than high school graduate. ^c^Model was adjusted for age at cohort entry and sex (as strata variables), marital status, smoking, body mass index (calculated as weight in kilograms divided by height in meters squared), vigorous physical activity, coffee intake, alcohol intake, total energy intake, percent energy from fat, and preexisting chronic disease. ^d^Native Hawaiian participants living in California are not shown owing to small numbers (160 individuals with 22 cardiovascular disease deaths).

**Figure 3.**
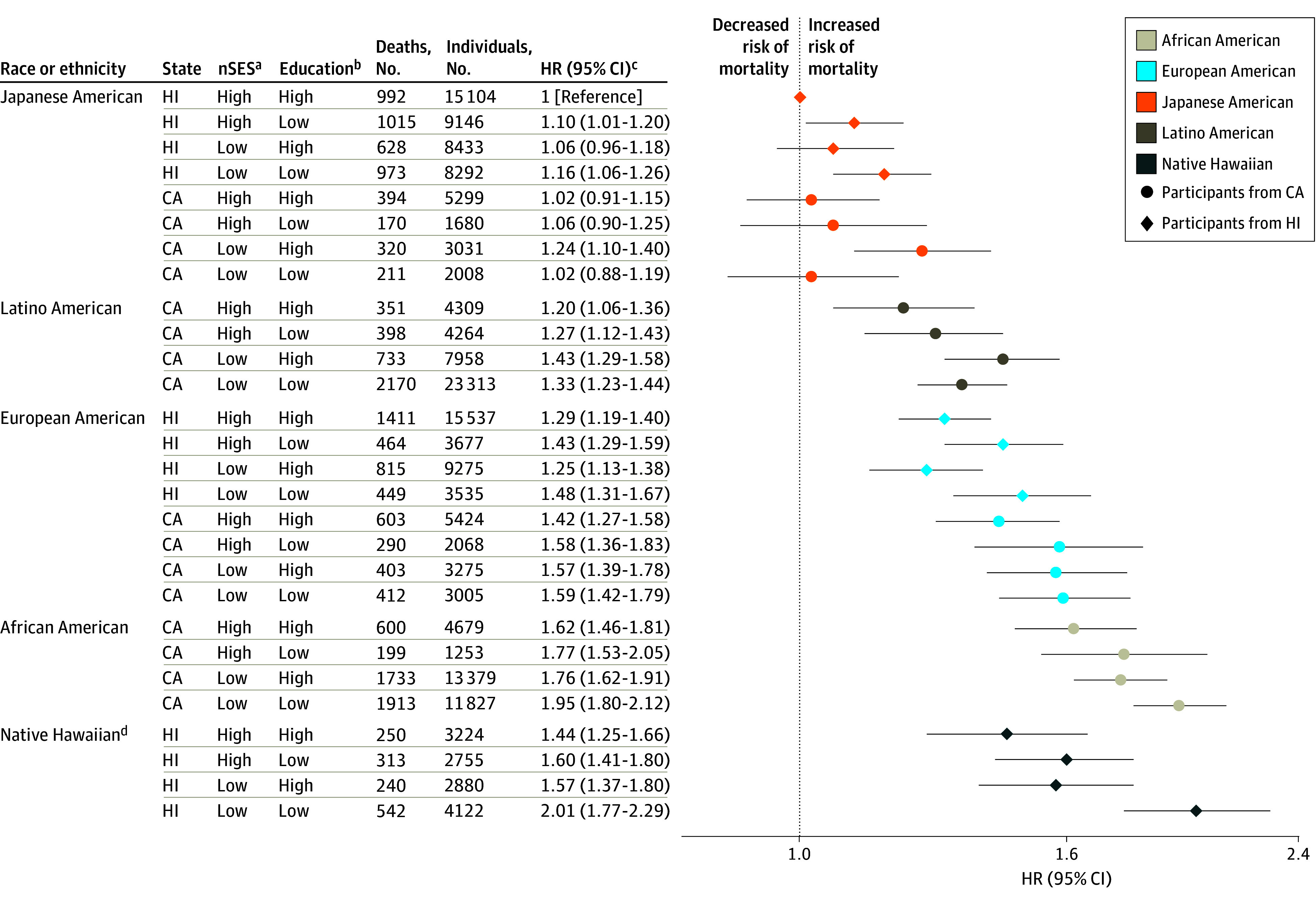
Joint Associations of Factors With Cancer Mortality Joint associations of race, ethnicity, state of residence, neighborhood socioeconomic status (nSES), and education with mortality are shown. ^a^Low nSES was defined as quintiles 1 to 3 and high nSES as quintiles 4 to 5. ^b^Low education was defined as high school graduate or less and high education as greater than high school graduate. ^c^Model was adjusted for age at cohort entry and sex (as strata variables), marital status, smoking, body mass index (calculated as weight in kilograms divided by height in meters squared), vigorous physical activity, coffee intake, alcohol intake, total energy intake, percent energy from fat, and preexisting chronic disease. ^d^Native Hawaiian participants living in California are not shown owing to small numbers (160 individuals with 16 cancer deaths).

Outcomes for CVD-specific mortality followed similar patterns to those for all-cause mortality (Figure 2). Native Hawaiian participants in Hawaii with low nSES and low education had the highest CVD mortality HR (HR, 2.92; 95% CI, 2.60-3.27). For cancer-specific mortality (Figure 3), HRs were highest for Native Hawaiian participants in Hawaii (HR, 2.01; 95% CI, 1.77-2.29) and African American participants in California (HR, 1.95; 95% CI, 1.80-2.12) with low nSES and low education. Among Japanese American participants in California and Latino American participants in California, those with low nSES and high education had the highest cancer-specific mortality HRs (1.24; 95% CI, 1.10-1.40 and 1.43; 95% CI, 1.29-1.58, respectively).

**Figure 4.**
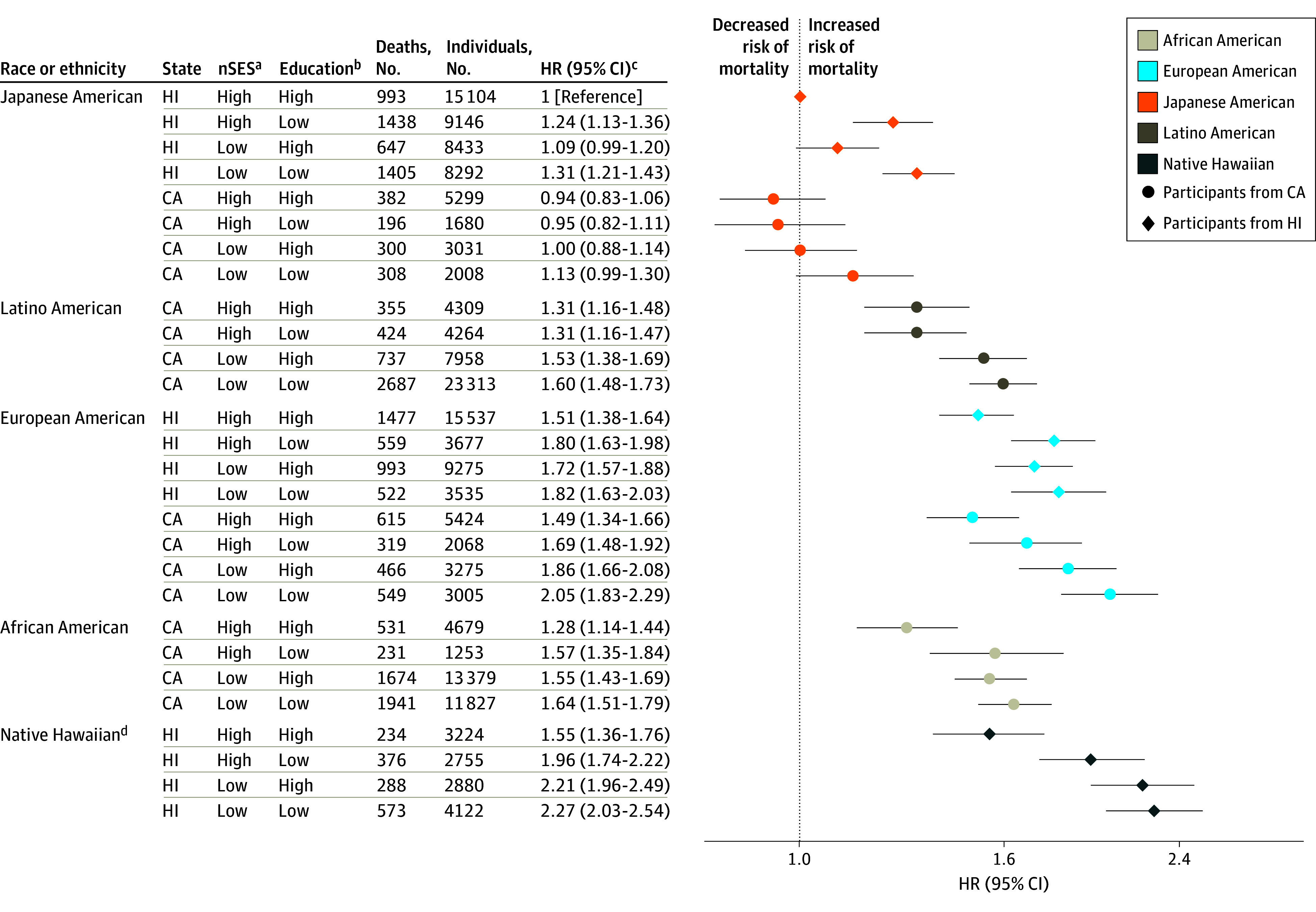
Joint Associations of Factors With Non–cardiovascular Disease and Noncancer Mortality Joint associations of race, ethnicity, state of residence, neighborhood socioeconomic status (nSES), and education with mortality are shown. ^a^Low nSES was defined as quintiles 1 to 3 and high nSES as quintiles 4 to 5. ^b^Low education was defined as high school graduate or less and high education as greater than high school graduate. ^c^Model was adjusted for age at cohort entry and sex (as strata variables), marital status, smoking, body mass index (calculated as weight in kilograms divided by height in meters squared), vigorous physical activity, coffee intake, alcohol intake, total energy intake, percent energy from fat, and preexisting chronic disease. ^d^Native Hawaiian participants living in California are not shown owing to small numbers (160 individuals with 15 non–cardiovascular disease and noncancer deaths).

Outcomes for mortality from causes other than CVD or cancer are presented in Figure 4. There were 993 such deaths among the reference group (6.6%). Native Hawaiian participants in Hawaii with low nSES and low education had the highest HR (2.27; 95% CI, 2.03-2.54). In contrast to the patterns seen for all-cause, CVD-specific, and cancer-specific mortality, HRs for non-CVD and noncancer mortality were higher for European American participants with low nSES and low education in Hawaii (HR, 1.82; 95% CI, 1.63-2.03) and California (HR, 2.05; 95% CI, 1.83-2.29) than for African American participants in California with low nSES and low education (HR, 1.64; 95% CI, 1.51-1.79).

Results from a minimal model adjusting for age at cohort entry and sex had similar patterns of associations by exposure category as those found in the multivariable model after adjusting for all confounders (eTable 3 in the Supplement). Similar patterns of associations with all-cause mortality were observed for men and women, although HRs were larger for women compared with men (eFigure 5 in the Supplement). After excluding participants with preexisting chronic conditions at baseline, HR estimates were smaller for all-cause mortality, although patterns of associations by the joint exposure variable were similar to those in the overall study population (eTable 4 in the Supplement).

## Discussion

The objective of this cohort study was to jointly consider the association of race, ethnicity, state of residence, nSES, and education with all-cause and cause-specific mortality for participants in the MEC, especially for groups often underrepresented in previous epidemiologic research. We initially hypothesized that nSES and education would further delineate racial and ethnic inequities in mortality. Using our analytic methods to test this hypothesis, we found a wide range of statistically significant outcomes in mortality across groups defined jointly by race, ethnicity, state of residence, nSES, and education in the MEC. Compared with Japanese American participants in Hawaii with high nSES and high education, Native Hawaiian participants in Hawaii with low nSES and low education experienced a 2.4-fold increase in all-cause mortality, and African American and European American participants in California with low nSES and low education experienced a 2-fold increase in all-cause mortality. For most groupings of race, ethnicity, and state of residence, mortality ranged from high to low in the following order: low nSES and low education, low nSES and high education, high nSES and low education, and high nSES and high education. The exception to this pattern was seen among Japanese American participants in Hawaii and European American participants in Hawaii, for whom mortality HRs were higher for those with high nSES and low education vs those with low nSES and high education. The strongest justification for the utility of joint analysis was found in the analysis among African American and Native Hawaiian participants; these groups had near equal all-cause mortality HRs overall, but in joint analysis with state, nSES, and education, Native Hawaiian participants in Hawaii with low nSES and low education had higher mortality HRs compared with African American participants in California with low nSES and low education. For CVD-specific and cancer-specific mortality, patterns were similar to those found for all-cause mortality, although HRs were generally larger for CVD-specific mortality and smaller for cancer-specific mortality.

Although joint associations of nSES and individual SES with all-cause mortality have been investigated in several US studies,^11,12,13,14,15,16^ only 3 studies sampled from the general population and had racially and ethnically diverse populations. In the Southern Community Cohort Study (SCCS),^13^ participants with low nSES and low individual SES (measured by annual household income) had the highest all-cause mortality HRs, and HRs were equivalent for African American and White participants. In the MEC, all-cause mortality HRs were higher for low nSES and low education vs high nSES and high education in each race, ethnicity, and state of residence strata, with HRs for African American participants higher than HRs for European American participants in Hawaii and similar to HRs for European American participants in California. In the Atherosclerosis Risk in Communities (ARIC) study^16^ and a study using data from the National Health Interview Survey (NHIS),^11^ mortality rates were highest for those with low nSES and low individual SES (measured by personal income in ARIC and household income to needs ratio in NHIS) and lowest for those with high nSES and high individual SES. Furthermore, mortality rates were higher for African American participants than White participants, which is in agreement with findings in the MEC for these 2 groups. Among Mexican American participants who completed the NHIS, the highest mortality rates were found for those with high nSES and low individual SES, and individual SES was associated with mortality rates only among participants with high nSES. In contrast, among Latino American participants in California in the MEC (who were predominantly Mexican American^34^), individual SES was not associated with mortality within high or low nSES groups. It is difficult to directly compare our findings with findings from these prior studies because we used education as our measure of individual SES, whereas prior studies used income. Although different measures of individual SES (income, wealth, occupation, and education being the most common) are correlated, they likely operate under different mechanisms in their associations with health and may not be equivalent.^35^

Studied separately, nSES and individual SES have been shown to be directly proportional to life expectancy.^7,17,18,36,37,38^ nSES is associated with health through a multitude of stressors that accumulate over an individual’s lifetime.^36^ Neighborhoods lower on the SES scale encompass attributes that are more detrimental to health and well-being. These attributes include increased access to alcohol and tobacco products, as well as poorer nutrition owing to lack of availability of nutrient-dense foods and abundance of lower-quality food.^7,36^ Furthermore, lack of recreational facilities and high crime rates limit physical activity opportunities in poorer neighborhoods, presenting challenges to health-promoting behaviors.^7,36^ Areas of low nSES also tend to lack quality health care facilities.^7^ Finally, compared with areas of high nSES, areas of low nSES may have increased levels of environmental pollutants.^8^

Education is associated with more occupational prospects and higher income potential.^18^ However, income disparities exist by race and ethnicity for every level of education, with African American and Latino American individuals earning significantly less than European American individuals.^7^ Higher educational attainment and personal income have been associated with increased knowledge of and access to health resources and adoption of healthy behaviors.^37^ Healthy lifestyle choices are associated with more favorable prognoses and longer survival after diagnosis of major chronic diseases. Higher education is also associated with increased autonomy and economic independence, which are associated with improved self-esteem. In turn, this sets up a possible pathway through which stress may be reduced, thereby improving mortality rates.^39,40^

Variation in SES by race and ethnicity is a product of historical racial and ethnic segregation and other forms of structural racism.^7,8,41^ Although we did not assess measures of segregation or structural racism, we found variation in nSES and education by race and ethnicity. Accumulative stressors for residents in poorer neighborhoods or for individuals with less educational attainment can be associated with adverse physiological changes and health behaviors over their lifetime, which can result in shorter life expectancy.^7,9^

To our knowledge, our study is the largest prospective analysis with the longest follow-up of the joint associations of nSES and individual SES with mortality in a racially and ethnically diverse study population that included a large number of underrepresented, understudied, and economically marginalized participants. We were well-powered to study the important associations of race, ethnicity, state of residence, nSES, and education with mortality. Our participants were sampled from 2 large and diverse states and resided in neighborhoods with a wide range of exposure assessment. Prior work found similar distributions of educational levels between the cohort sample and 1990 census data for Hawaii and Los Angeles County, suggesting that our SES findings may be broadly generalizable to these populations.^23^ Cancer incidence rates in the MEC also closely mirrored those of Hawaii and Southern California by race and ethnic group for men and women.

Several strengths support the internal validity of our results. We used high-quality questionnaire data on a comprehensive set of individual-level risk factors to account for confounding. MEC data agree on overlapping variables (eg, race and ethnicity, age, and weight and height) when linked to external secondary sources (eg, a tumor registry or hospital discharge diagnosis files). Additionally, our assessment of diet^42^ and physical activity^43^ have been previously validated, and all baseline addresses represented deliverable mailing addresses. Quintile distributions for nSES were specific to California and Hawaii separately. This suggests that differences in certain economic characteristics, such as cost of living, between states were unlikely to bias results. We used information on educational attainment, which is a well-validated measure associated with individual SES in health research studies.^18^ Finally, a long follow-up period of up to 20 years included a substantial number of deaths, with all deaths in the US identified via the US National Death Index.

### Limitations

There are several limitations to our study. As with any observational study, we cannot exclude the possibility of residual or unmeasured confounding. We used census block groups based on administrative boundaries to define geographical neighborhoods. We recognize that participants’ self-reported perceived measures of neighborhood may be more representative of participants’ lived experience than geospatial measures.^36^ Furthermore, data on individual-level income or wealth, often used in the literature to assess individual SES,^35^ were not available. Therefore, we were unable to explore associations for measures of individual SES other than education. During up to 20 years of follow-up, approximately 79% of MEC participants remained at the same level of nSES between baseline and death or censor. Although nSES can fluctuate during follow-up, we chose to use baseline nSES to allow for sufficient time for this exposure to be associated with mortality because baseline exposures more closely capture exposures relevant to the latency period for disease development and mortality outcomes.

## Conclusions

The combination of race, ethnicity, state of residence, nSES, and education allowed us to identify patterns of associations with mortality not otherwise discernable had we studied these factors separately. These findings suggest that examining social inequities in health by jointly considering race, ethnicity, and SES is critical because these factors do not appear to be independent; instead, they coexist and intersect in their associations with health. Quantitative population health studies, such as ours, may contribute to developing policies and interventions that advance social justice and health equity among multiple marginalized social groups.^44^ Certain populations, such as African American and Native Hawaiian groups, have had a long history of premature mortality in the US. In an effort to achieve better health equity for these and other underserved populations at increased risk of poor health outcomes, our findings suggest that future studies should aim to examine place of residence and its economic milieu using measures of education, income, social identity, social stressors, and individual-level risk factors and work closely with community stakeholders in translating findings into interventions that may ameliorate health inequities.

## Notes

Supplement. **eFigure 1.** Recruitment and Enrollment in the Multiethnic Cohort **eFigure 2.** Self-reported Race and Ethnicity Categories in the Multiethnic Cohort Baseline Questionnaire **eTable 1.** US Census Block Group Characteristics by Neighborhood Socioeconomic Status in Los Angeles County and Hawaii in 1990 **eFigure 3.** Distribution of Baseline Neighborhood Socioeconomic Status by Race, Ethnicity, and State of Residence in the Multiethnic Cohort **eFigure 4.** Distribution of Baseline Education by Race, Ethnicity, and State of Residence in the Multiethnic Cohort **eTable 2.** Association of Race and Ethnicity With All-Cause Mortality Among 182 912 Participants in the Multiethnic Cohort **eTable 3.** Joint Associations of Factors With Mortality Adjusted for Age at Cohort Entry and Sex Among 182 912 Participants in the Multiethnic Cohort **eFigure 5.** Joint Associations of Factors With All-Cause Mortality by Sex Among 182 912 Participants in the Multiethnic Cohort **eTable 4.** Joint Associations of Factors With All-Cause Mortality Among 88 665 Participants Without Previous Chronic Illness in the Multiethnic Cohort
