# Errors in Table Data

**DOI:** 10.1001/jamanetworkopen.2024.43737

**Published:** 2024-10-15

In the Original Investigation titled “Joint Associations of Race, Ethnicity, and Socioeconomic Status With Mortality in the Multiethnic Cohort Study,”^1^ published April 11, 2022, counts and percentages for the columns “Japanese American” and “Latino American” were reversed for the variable “Joint nSES and education” in the Table and there were errors in the count of Native Hawaiian individuals living in California in footnotes for all Figures and eFigure 5 and eTable 4 in the Supplement. The article has been corrected.^1^
