# Supplementary material for: Joint Associations of Race, Ethnicity, and Socioeconomic Status With Mortality in the Multiethnic Cohort Study
Source: JAMA Netw Open. 2022 Apr 11;5(4):e226370. doi: 10.1001/jamanetworkopen.2022.6370 (PMC9002338; doi:10.1001/jamanetworkopen.2022.6370)
Supplement: Supplement. — eFigure 1. Recruitment and Enrollment in the Multiethnic Cohort eFigure 2. Self-reported Race and Ethnicity Categories in the Multiethnic Cohort Baseline Questionnaire eTable 1. US Census Block Group Characteristics by Neighborhood Socioeconomic Status in Los Angeles County and Hawaii in 1990 eFigure 3. Distribution of Baseline Neighborhood Socioeconomic Status by Race, Ethnicity, and State of Residence in the Multiethnic Cohort eFigure 4. Distribution of Baseline Education by Race, Ethnicity, and State of Residence in the Multiethnic Cohort eTable 2. Association of Race and Ethnicity With All-Cause Mortality Among 182 912 Participants in the Multiethnic Cohort eTable 3. Joint Associations of Factors With Mortality Adjusted for Age at Cohort Entry and Sex Among 182 912 Participants in the Multiethnic Cohort eFigure 5. Joint Associations of Factors With All-Cause Mortality by Sex Among 182 912 Participants in the Multiethnic Cohort eTable 4. Joint Associations of Factors With All-Cause Mortality Among 88 665 Participants Without Previous Chronic Illness in the Multiethnic Cohort [file jamanetwopen-e226370-s001.pdf]

Supplemental Online Content

Sangaramoorthy M, Shariff-Marco S, Conroy SM, et al. Joint associations of race, ethnicity, and socioeconomic status with mortality in the Multiethnic Cohort Study. *JAMA Netw Open*. 2022;5(4):e226370. doi:10.1001/jamanetworkopen.2022.6370

eFigure 1. Recruitment and Enrollment in the Multiethnic Cohort

eFigure 2. Self-reported Race and Ethnicity Categories in the Multiethnic Cohort Baseline Questionnaire

eTable 1. US Census Block Group Characteristics by Neighborhood Socioeconomic Status in Los Angeles County and Hawaii in 1990

eFigure 3. Distribution of Baseline Neighborhood Socioeconomic Status by Race, Ethnicity, and State of Residence in the Multiethnic Cohort

eFigure 4. Distribution of Baseline Education by Race, Ethnicity, and State of Residence in the Multiethnic Cohort

eTable 2. Association of Race and Ethnicity With All-Cause Mortality Among 182 912 Participants in the Multiethnic Cohort

eTable 3. Joint Associations of Factors With Mortality Adjusted for Age at Cohort Entry and Sex Among 182 912 Participants in the Multiethnic Cohort

eFigure 5. Joint Associations of Factors With All-Cause Mortality by Sex Among 182 912 Participants in the Multiethnic Cohort

eTable 4. Joint Associations of Factors With All-Cause Mortality Among 88 665 Participants Without Previous Chronic Illness in the Multiethnic Cohort

This supplemental material has been provided by the authors to give readers additional information about their work.

© 2022 Sangaramoorthy M et al. *JAMA Network Open.*

eFigure 1. Recruitment and Enrollment in the Multiethnic Cohort

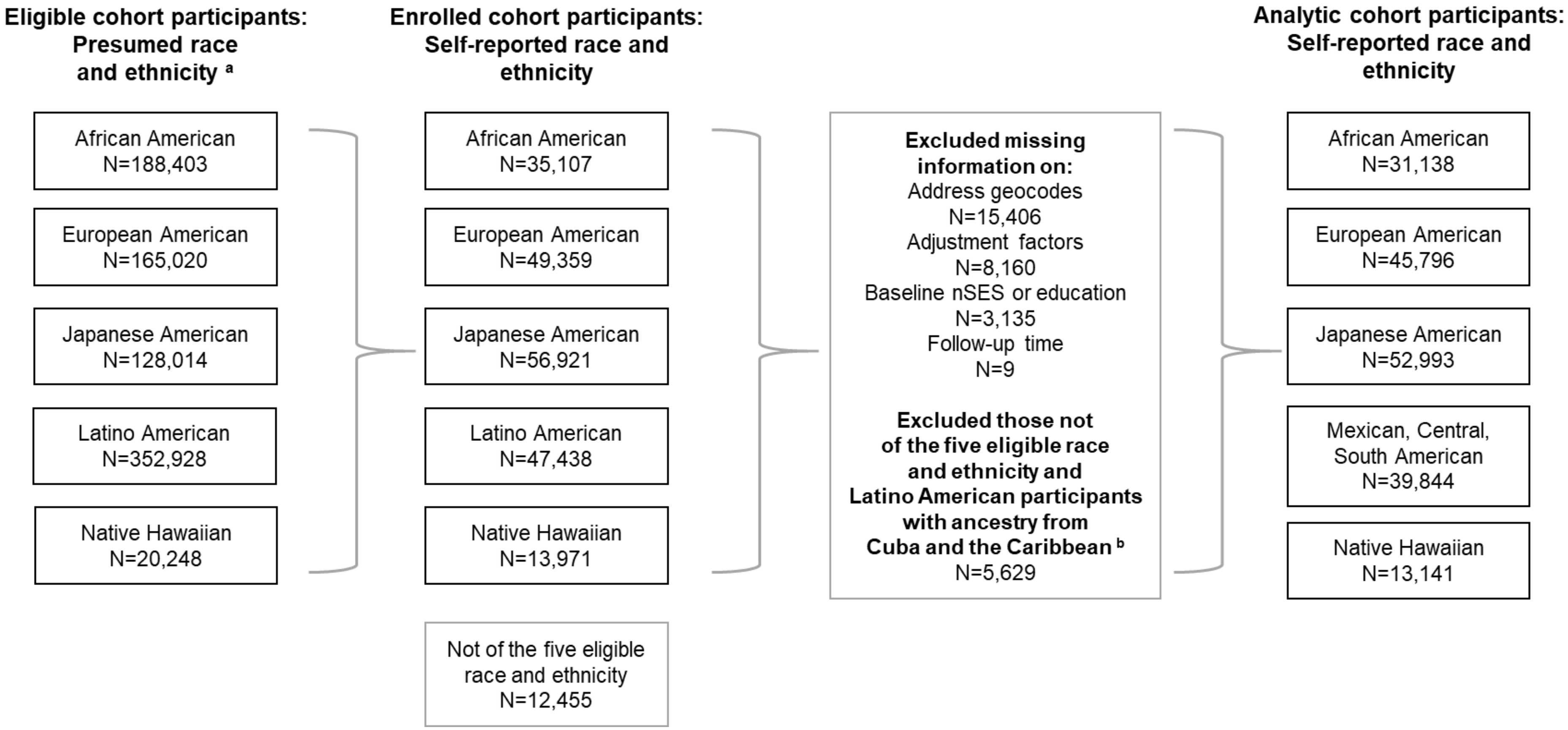

[a]Identified primarily through driver license files, supplemented with voter registration files in Hawaii and Health Care Financing Administration files in California. Since driver license files did not include racial or ethnic information, presumptive race and ethnicity based on surname and zip code were used for eligibility selection purposes by trained study staff. [b]Includes 4,049 Asian American participants (3,073 Filipino American, 178 Chinese American, 55 Korean American), 1,580 Cuban/Caribbean Latino American participants with inadequate dietary assessment, and 743 participants of other or unknown race and ethnicity.

© 2022 Sangaramoorthy M et al. *JAMA Network Open.*

eFigure 2. Self-reported Race and Ethnicity Categories in the Multiethnic Cohort Baseline Questionnaire

7. WHAT IS YOUR ETHNIC OR RACIAL BACKGROUND? ***(Mark all that apply)***

- ○ Black or African-American
- ○ Chinese
- ○ Filipino
- ○ Hawaiian
- ○ Japanese (includes Okinawan)
- ○ Korean
- ○ Mexican or other Hispanic
- ○ White or Caucasian
- ○ Other: *(write in)* ☐

© 2022 Sangaramoorthy M et al. *JAMA Network Open.*

eTable 1. US Census Block Group Characteristics by Neighborhood Socioeconomic Status in Los Angeles County and Hawaii in 1990

	**Los Angeles County**					**Hawaii**				
**nSES quintile**	**Q1**	**Q2**	**Q3**	**Q4**	**Q5**	**Q1**	**Q2**	**Q3**	**Q4**	**Q5**
% Without high school diploma	61.2	39.3	25.2	15.8	8.3	36.6	27.9	21.7	16.1	9.5
% With high school diploma	18.6	23.3	25	23.4	16.1	31.3	32	29.1	28.6	22.1
% With college degree	20.2	37.4	49.8	60.9	75.6	32.1	40.2	49.1	55.4	68.4
Median household income ($)	20,785	29,907	35,741	42,865	59,956	22,552	32,788	38,226	44,615	59,782
% Below 200% of federal poverty line	64.1	40.5	26.1	16.6	9.8	46.4	27.8	21.1	15.9	7.6
% Blue-collar workers (age $\geq$ 16 yr)	70.5	56.12	47.06	39.6	30.86	60.6	58.1	49.1	45.9	37.0
% Unemployed (age $\geq$ 16 yr)	13.8	9	6.3	4.5	3.3	6.8	4.1	3.2	3	2.3
Median gross rent per housing unit ($)	509	613	668	759	950	372	569	643	747	1001
Median home value per single family home ($)	128,800	165,150	195,500	244,600	409,450	89,450	162,500	217,250	255,700	350,000

Abbreviations: nSES, neighborhood socioeconomic status; Q, quintile

© 2022 Sangaramoorthy M et al. *JAMA Network Open.*

eFigure 3. Distribution of Baseline Neighborhood Socioeconomic Status by Race, Ethnicity, and State of Residence in the Multiethnic Cohort

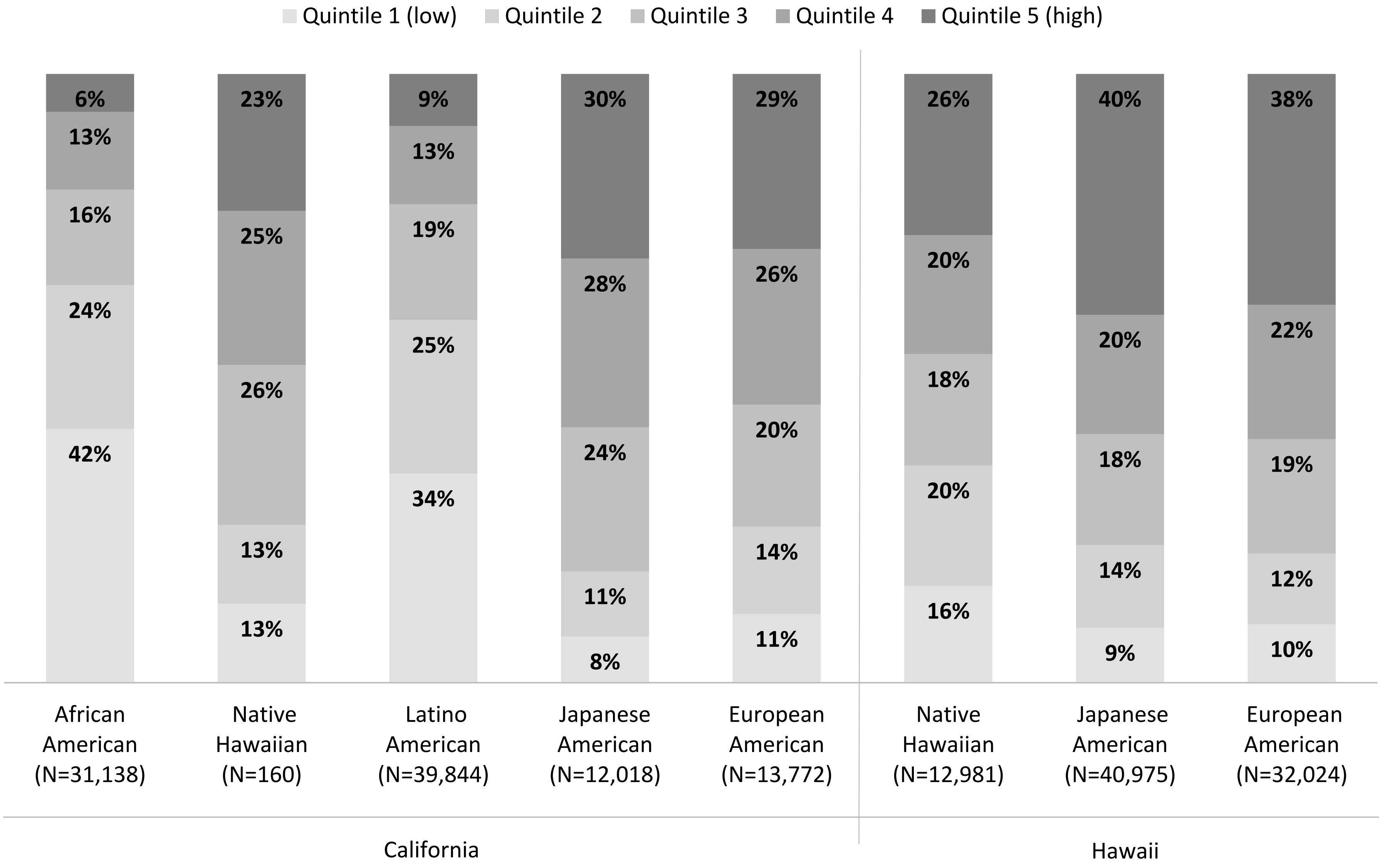

© 2022 Sangaramoorthy M et al. *JAMA Network Open.*

eFigure 4. Distribution of Baseline Education by Race, Ethnicity, and State of Residence in the Multiethnic Cohort

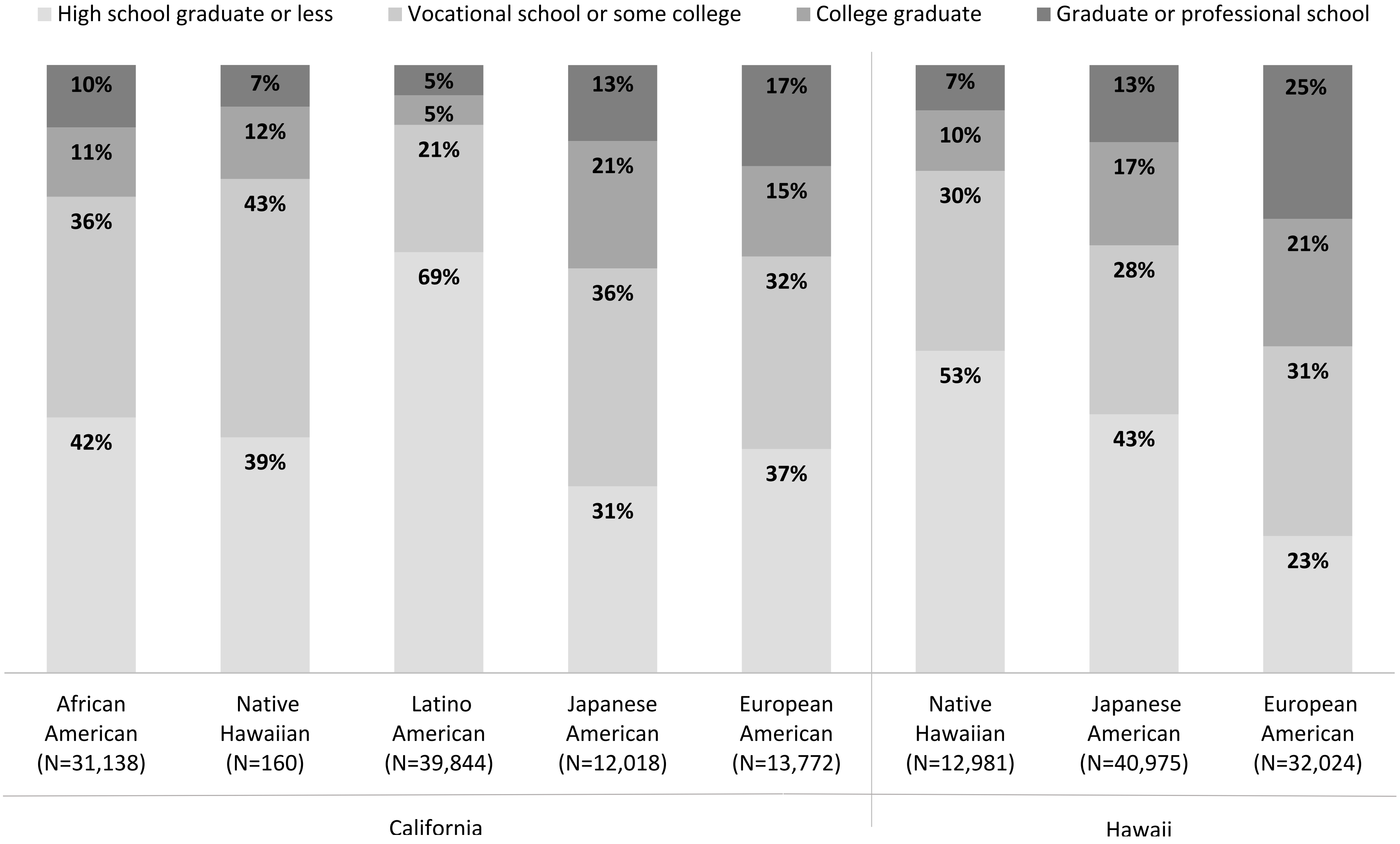

© 2022 Sangaramoorthy M et al. *JAMA Network Open.*

eTable 2. Association of Race and Ethnicity With All-Cause Mortality Among 182 912 Participants in the Multiethnic Cohort

Race and ethnicity	Deaths N	HR (95% CI)[a]
Japanese American	15,956	1.00
Latino American	12,485	1.24 (1.21-1.27)
European American	15,531	1.42 (1.39-1.46)
African American	14,975	1.93 (1.89-1.98)
Native Hawaiian	4,542	1.96 (1.90-2.03)

Abbreviations: HR, hazard ratio; CI, confidence interval.
[a] Model was adjusted for age at cohort entry and sex (as strata variables), marital status, smoking, BMI, vigorous physical activity, coffee intake, alcohol intake, total energy intake, and percent energy from fat.

© 2022 Sangaramoorthy M et al. *JAMA Network Open.*

eTable 3. Joint Associations of Factors With Mortality Adjusted for Age at Cohort Entry and Sex Among 182 912 Participants in the Multiethnic Cohort

				All-cause mortality		Cardiovascular Disease mortality		Cancer mortality		Non-cardiovascular disease/cancer mortality	
**Race and ethnicity**	**State**	**nSES[a]**	**Education[b]**	**Deaths N**	**HR (95% CI)[c]**	**Deaths N**	**HR (95% CI)[c]**	**Deaths N**	**HR (95% CI)[c]**	**Deaths N**	**HR (95% CI)[c]**
Japanese American	HI	High	High	2,870	Reference	884	Reference	992	Reference	993	Reference
		High	Low	3,656	1.26 (1.20-1.32)	1,203	1.26 (1.15-1.38)	1,015	1.19 (1.09-1.29)	1,438	1.34 (1.22-1.47)
		Low	High	1,919	1.15 (1.09-1.21)	640	1.24 (1.11-1.38)	628	1.10 (0.99-1.22)	647	1.12 (1.01-1.23)
		Low	Low	3,750	1.40 (1.33-1.47)	1,370	1.54 (1.41-1.70)	973	1.24 (1.13-1.35)	1,405	1.42 (1.31-1.54)
	CA	High	High	1,200	1.02 (0.95-1.09)	420	1.13 (1.01-1.28)	394	1.01 (0.90-1.13)	382	0.93 (0.82-1.05)
		High	Low	640	1.22 (1.12-1.32)	272	1.58 (1.37-1.82)	170	1.10 (0.93-1.30)	196	1.01 (0.87-1.18)
		Low	High	1,007	1.29 (1.20-1.38)	385	1.52 (1.34-1.73)	320	1.30 (1.15-1.47)	300	1.08 (0.95-1.22)
		Low	Low	914	1.40 (1.29-1.52)	394	1.82 (1.59-2.07)	211	1.12 (0.96-1.30)	308	1.28 (1.11-1.47)
Latino American	CA	High	High	1,092	1.20 (1.12-1.28)	385	1.36 (1.20-1.54)	351	1.10 (0.97-1.25)	355	1.15 (1.02-1.29)
		High	Low	1,310	1.30 (1.22-1.39)	488	1.55 (1.37-1.75)	398	1.20 (1.06-1.35)	424	1.19 (1.07-1.34)
		Low	High	2,299	1.48 (1.40-1.57)	827	1.73 (1.57-1.92)	733	1.35 (1.22-1.48)	737	1.40 (1.27-1.54)
		Low	Low	7,784	1.57 (1.50-1.64)	2,925	1.90 (1.74-2.06)	2,170	1.29 (1.19-1.39)	2,687	1.56 (1.45-1.68)
European American	HI	High	High	4,205	1.44 (1.36-1.52)	1,311	1.43 (1.29-1.58)	1,411	1.42 (1.31-1.54)	1,477	1.46 (1.33-1.60)
		High	Low	1,502	1.88 (1.76-2.01)	479	1.88 (1.69-2.09)	464	1.79 (1.61-1.99)	559	1.98 (1.79-2.19)
		Low	High	2,657	1.62 (1.52-1.72)	848	1.66 (1.50-1.84)	815	1.45 (1.31-1.60)	993	1.75 (1.60-1.93)
		Low	Low	1,500	2.07 (1.94-2.22)	529	2.31 (2.05-2.59)	449	1.88 (1.65-2.13)	522	2.07 (1.86-2.30)
	CA	High	High	1,829	1.51 (1.40-1.63)	610	1.60 (1.43-1.80)	603	1.51 (1.34-1.70)	615	1.43 (1.28-1.60)
		High	Low	953	1.96 (1.80-2.14)	343	2.22 (1.96-2.52)	290	1.88 (1.60-2.21)	319	1.83 (1.60-2.08)
		Low	High	1,342	2.01 (1.88-2.16)	473	2.26 (2.02-2.54)	403	1.82 (1.60-2.06)	466	1.98 (1.77-2.23)
		Low	Low	1,543	2.36 (2.20-2.53)	582	2.80 (2.50-3.13)	412	1.96 (1.73-2.21)	549	2.35 (2.11-2.63)
African American	CA	High	High	1,802	1.66 (1.55-1.77)	670	1.93 (1.73-2.16)	600	1.73 (1.55-1.92)	531	1.36 (1.21-1.53)
		High	Low	674	2.10 (1.93-2.30)	242	2.30 (1.97-2.67)	199	2.05 (1.78-2.37)	231	2.00 (1.72-2.31)
		Low	High	5,724	2.26 (2.15-2.37)	2,310	2.93 (2.68-3.20)	1,733	2.06 (1.90-2.23)	1,674	1.87 (1.73-2.03)
		Low	Low	6,775	2.71 (2.59-2.84)	2,911	3.63 (3.33-3.96)	1,913	2.41 (2.22-2.61)	1,941	2.18 (2.01-2.36)
Native Hawaiian[d]	HI	High	High	755	1.69 (1.56-1.83)	271	2.02 (1.75-2.33)	250	1.51 (1.31-1.74)	234	1.57 (1.39-1.79)
		High	Low	1,106	2.30 (2.16-2.46)	416	2.79 (2.44-3.18)	313	1.89 (1.67-2.13)	376	2.29 (2.03-2.57)

© 2022 Sangaramoorthy M et al. *JAMA Network Open.*

		Low	High	795	2.21 (2.02-2.40)	266	2.50 (2.16-2.89)	240	1.77 (1.54-2.03)	288	2.41 (2.14-2.72)
		Low	Low	1,831	2.87 (2.65-3.11)	715	3.65 (3.24-4.11)	542	2.41 (2.11-2.74)	573	2.66 (2.37-2.98)

Abbreviations: nSES, neighborhood socioeconomic status; HR, hazard ratio; CI, confidence interval.
[a] Low nSES was defined as quintiles 1 to 3 and high nSES defined as quintiles 4 to 5.
[b] Low education was defined as high school graduate or less and high education defined as greater than high school graduate.
[c] Model was adjusted for age at cohort entry and sex as strata variables.
[d] Native Hawaiian participants in California not shown owing to small numbers (160 individuals with 55 all-cause deaths, 22 cardiovascular disease deaths, 16 cancer deaths,15 noncardiovascular disease and noncancer deaths).

© 2022 Sangaramoorthy M et al. *JAMA Network Open.*

eFigure 5. Joint Associations of Factors With All-Cause Mortality by Sex Among 182 912 Participants in the Multiethnic Cohort

Race/ethnicity	State/nSES[a]/Education[b]	A) Females: Deaths N	A) Females: HR (95% CI)[c]	B) Males: Deaths N	B) Males: HR (95% CI)[c]
Japanese American	HI / high / high	1,110	Reference	1,760	Reference
	HI / high / low	1,626	1.18 (1.10-1.27)	2,030	1.18 (1.10-1.26)
	HI / low / high	722	1.08 (0.99-1.17)	1,197	1.15 (1.06-1.24)
	HI / low / low	1,677	1.34 (1.25-1.44)	2,073	1.28 (1.20-1.37)
	CA / high / high	419	1.06 (0.94-1.18)	781	1.02 (0.94-1.10)
	CA / high / low	320	1.21 (1.08-1.36)	320	1.13 (1.00-1.28)
	CA / low / high	374	1.25 (1.10-1.41)	633	1.18 (1.08-1.29)
	CA / low / low	431	1.30 (1.16-1.46)	483	1.25 (1.11-1.40)
Latino American	CA / high / high	320	1.38 (1.23-1.56)	772	1.27 (1.17-1.38)
	CA / high / low	581	1.39 (1.26-1.54)	729	1.39 (1.27-1.52)
	CA / low / high	900	1.73 (1.59-1.89)	1,399	1.45 (1.35-1.56)
	CA / low / low	3,628	1.64 (1.54-1.75)	4,156	1.51 (1.42-1.60)
European American	HI / high / high	1,704	1.53 (1.42-1.65)	2,501	1.37 (1.28-1.46)
	HI / high / low	697	1.63 (1.49-1.79)	805	1.65 (1.51-1.80)
	HI / low / high	1,099	1.62 (1.50-1.76)	1,558	1.46 (1.36-1.57)
	HI / low / low	643	1.71 (1.55-1.88)	857	1.79 (1.64-1.95)
	CA / high / high	947	1.62 (1.48-1.78)	882	1.42 (1.30-1.54)
	CA / high / low	622	1.82 (1.65-2.01)	331	1.63 (1.44-1.85)
	CA / low / high	772	2.02 (1.84-2.21)	570	1.58 (1.43-1.75)
	CA / low / low	990	2.08 (1.89-2.28)	553	1.79 (1.62-1.98)
African American	CA / high / high	907	1.50 (1.37-1.64)	895	1.51 (1.39-1.64)
	CA / high / low	361	1.58 (1.39-1.80)	313	1.71 (1.49-1.96)
	CA / low / high	3,438	1.87 (1.75-2.00)	2,286	1.75 (1.63-1.87)
	CA / low / low	3,995	2.07 (1.94-2.22)	2,780	1.91 (1.79-2.04)
Native Hawaiian[d]	HI / high / high	316	1.63 (1.43-1.86)	439	1.57 (1.42-1.74)
	HI / high / low	547	1.97 (1.79-2.17)	559	1.87 (1.69-2.07)
	HI / low / high	392	2.06 (1.84-2.31)	403	1.84 (1.65-2.05)
	HI / low / low	969	2.45 (2.25-2.67)	862	2.26 (2.02-2.51)

A) Females

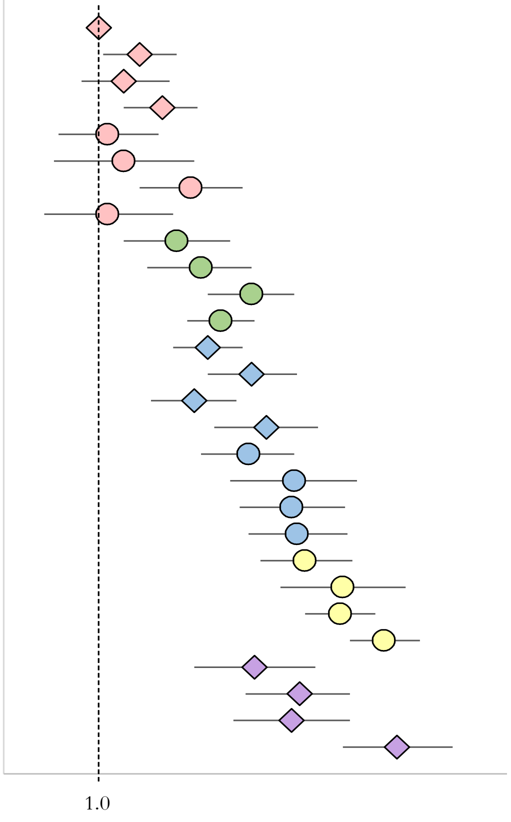

B) Males

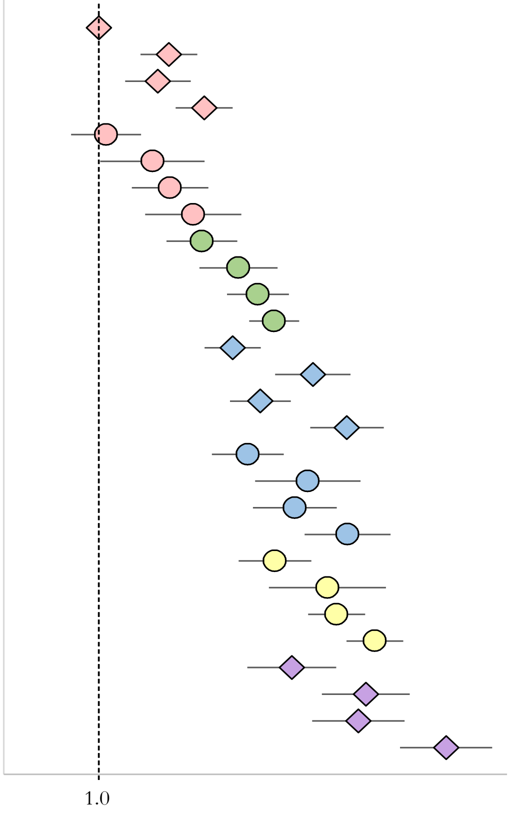

Abbreviations: nSES, neighborhood socioeconomic status; HR, hazard ratio; CI, confidence interval; HI, Hawaii; CA, California. Joint associations of race, ethnicity, state of residence, neighborhood socioeconomic status (nSES), and education with mortality adjusted for age at cohort entry and sex are shown. Blue indicates European American participants; circles, participants from CA; diamonds, participants from HI; green, Latino American participants; HR, hazard ratio; orange, Japanese American participants; purple, Native Hawaiian participants; yellow, African American participants. [a] Low nSES was defined as quintiles 1 to 3 and high nSES defined as quintiles 4 to 5. [b] Low education was defined as high school graduate or less and high education defined as greater than high school graduate. [c] Model was adjusted for age at cohort entry (as a strata variable), marital status, smoking, BMI, vigorous physical activity, coffee intake, alcohol intake, total energy intake, percent energy from fat, and preexisting chronic disease. [d] Native Hawaiian participants in California not shown owing to small numbers (160 individuals with 55 deaths).

© 2022 Sangaramoorthy M et al. *JAMA Network Open.*

eTable 4. Joint Associations of Factors With All-Cause Mortality Among 88 665 Participants Without Previous Chronic Illness[a] in the Multiethnic Cohort

Race and ethnicity	State	nSES[b]	Education[c]	Deaths N	HR (95% CI)[d]
Japanese American	Hawaii	High	High	1,025	1.00
		High	Low	1,174	1.13 (1.03-1.24)
		Low	High	704	1.09 (0.99-1.20)
		Low	Low	1,210	1.29 (1.18-1.41)
	California	High	High	417	0.94 (0.84-1.06)
		High	Low	241	1.24 (1.08-1.44)
		Low	High	360	1.24 (1.10-1.41)
		Low	Low	313	1.22 (1.06-1.40)
Latino American	California	High	High	383	1.19 (1.06-1.35)
		High	Low	472	1.25 (1.11-1.41)
		Low	High	811	1.40 (1.27-1.54)
		Low	Low	2,537	1.39 (1.28-1.50)
European American	Hawaii	High	High	1,748	1.27 (1.17-1.39)
		High	Low	533	1.55 (1.39-1.72)
		Low	High	1,099	1.36 (1.23-1.50)
		Low	Low	515	1.54 (1.37-1.73)
	California	High	High	717	1.38 (1.25-1.53)
		High	Low	351	1.77 (1.53-2.04)
		Low	High	500	1.64 (1.47-1.84)
		Low	Low	506	1.88 (1.67-2.12)
African American	California	High	High	465	1.49 (1.33-1.66)
		High	Low	138	1.84 (1.51-2.23)
		Low	High	1,313	1.77 (1.61-1.95)
		Low	Low	1,364	1.97 (1.80-2.17)
Native Hawaiian[e]	Hawaii	High	High	223	1.41 (1.21-1.64)
		High	Low	304	1.85 (1.64-2.08)
		Low	High	247	1.87 (1.62-2.16)
		Low	Low	440	2.13 (1.87-2.43)

Abbreviations: nSES, neighborhood socioeconomic status; HR, hazard ratio; CI, confidence interval.
[a] Self-reported heart attack or angina, stroke, diabetes, or high blood pressure, and cancer that was self-reported or ascertained from tumor registries.
[b] Low nSES was defined as quintiles 1 to 3 and high nSES defined as quintiles 4 to 5.
[c] Low education was defined as high school graduate or less and high education defined as greater than high school graduate.
[d] Model was adjusted for age at cohort entry and sex (as strata variables), marital status, smoking, BMI, vigorous physical activity, coffee intake, alcohol intake, total energy intake, and percent energy from fat.
[e] Native Hawaiian participants in California not shown owing to small numbers (78 individuals with 16 deaths).

© 2022 Sangaramoorthy M et al. *JAMA Network Open.*
